# The decay-accelerating factor (CD55) in acute leukemia patients and its query implication in cancer pathogenesis

**DOI:** 10.1186/s43046-025-00299-7

**Published:** 2025-05-27

**Authors:** Hisham Abdelaziz, Mona Abdellateif, Ghada Elnaggar, Samar Kassem, Perihan Mohamed, Mohamed El Aziz, Khaled Abo-Aisha, Noha Farag, Noha Hassan

**Affiliations:** 1 Cairo University, Giza, Egypt; 2 Galala University, Suez, Egypt; 3 October 6 University, Giza, Egypt; 4 German University in Cairo, Cairo, Egypt

**Keywords:** Complement, MCRP, DAF, CD55, ALL, AML, ShRNA

## Abstract

**Background:**

The role of the complement system and its membrane-bound regulatory proteins (mCRPs) in the pathogenesis of cancer is still a debatable issue. The current study aimed to evaluate the role of the complement regulatory protein, the decay-accelerating factor (CD55), in the pathogenesis of acute leukemia.

**Methods:**

CD55 gene expression was assessed in the peripheral blood of 34 patients with acute myeloid leukemia (AML), 26 patients with acute lymphoblastic leukemia (ALL), and 30 healthy controls by qRT-PCR. Also, CD55 gene knockdown was performed in HSB-2 (ALL cell line) using customized short hairpin RNA (shRNA). Flowcytometric analysis was done to ensure successful transfection, and MTT assay was performed to evaluate the cell viability post-transfection and silencing of CD55.

**Results:**

There was a significant downregulation of CD55 in acute leukemia patients compared to the healthy controls (*p* < 0.001) with RQ values of AML, and ALL patients were 0.2499 ± 0.07427 and 0.2581 ± 0.09467, respectively. The MTT assay showed a significantly reduced viability of HSB-2 cells following posttranscriptional silencing of CD55 (*p* < 0.001) by 78.6% as compared to the non-transfected or mock-transfected cells. In the presence of human serum, there was a significant reduction in cell viability by 66.3% as compared to non-transfected controls (*p* = 0.01). Regarding the cells co-transfected with CD55 and CD46 silencing plasmids, cell viability was significantly decreased by 70.6% compared to non-transfected cells.

**Conclusion:**

CD55 was significantly downregulated in acute leukemia. However, its in vitro silencing showed significant reduction in cell viability, giving it a dual opposing role in cancer.

## Introduction

The complement system is an essential component of the innate immune system that can be found in both the plasm and as inactive cell surface proteins [1].

Many studies highlighted the important role of the complement system in suppressing tumor development through several mechanisms [2–4]. For example, the identification of specific proteins expressed on the surface of the neoplastic cells by the immunoglobulin-M (IgM) results in complement activation and consequently complement-mediated tumor cell lysis [4]. Others reported that complement activation results in the induction of apoptosis in the tumor cells [2, 3]. Though the complement system has a protective role against cancer initiation and progression, other studies reported that the complement system could promote tumor progression through the excessive inflammatory response induced by complement activation [5, 6].

Indeed, the complement system is continuously under control by regulatory proteins that protect against complement overactivation and cellular damage [7]. These regulatory proteins include the protectin (CD59) which inhibits cell lysis by suppressing the formation of the membrane attack complex (MAC) and preventing cell death. Another inhibitory protein is the membrane cofactor protein (MCP, CD46) which inhibits the C3b. Finally, it is the decay-accelerating factor (DAF, CD55) which destroys the C3 and C5 convertase, thus inhibiting the complement-mediated tumor cell lysis [8, 9]. The CD55 had been reported to have a regulatory function on the immune response to tumor cells by suppressing the adaptive immune response through the formation of a complex with CD97 [10, 11]. This complex acts as a costimulatory protein that induces naïve T-cell proliferation [12]. These naïve T cells function like T-regulatory cells (Tregs), which suppress the immune response against cancer, thus promoting its progression [13]. Moreover, the CD55 and CD97 complex increases the secretion of interleukin-10 (IL-10), granulocyte macrophage colony-stimulating factor (GM-CSF), in addition to the CD69 and CD25 markers [14]. The CD55 also suppresses the innate immune response through the inhibition of the natural killer (NK) cells [15]. Therefore, CD55 was investigated as a marker for tumor aggression in many cancers including colon [16], esophageal [17], breast [18], prostate [19], and gastric cancer [20].

On the other hand, CD55 was reported to work through a noncomplement-mediated or a noncanonical manner to promote tumor progression and metastasis [21]. Similarly, many studies suggested that CD55 activates intracellular signalling independent of CD97 complex formation [22]. It was found that CD55 can activate JNK/LCK signalling pathway in endometrioid tumors without CD97 activation [22].

Importantly, the expression of CD55 is regulated by many factors such as IL-1α, IL-1β, IL-4, tumor necrosis factor-α (TNF-α), interferon-γ (IFNγ), epidermal growth factor (EGF), and prostaglandin E2 which have a role in cancer pathogenesis as well [23, 24].

Acute leukemia is a malignant neoplasm that originates in the blood-forming cells in the bone marrow (Saygin BM). It is characterized by abnormal proliferation of the leukocytes [25]. Acute leukemias including acute myeloid leukemia (AML) and acute lymphoblastic leukemia (ALL) are considered life-threatening diseases in both children and adults [26]. The current study is designed to investigate the role of the membrane-bound complement regulatory protein (mCRP) CD55 in the pathogenesis of acute leukemia. This will be performed through the following: first is to assess the expression level of the DAF (CD55) gene in the peripheral blood (PB) of ALL and AML patients by real-time quantitative polymerase chain reaction (RT-qPCR) and the second is to investigate the effect of posttranscriptional knockdown of the CD55 gene using antisense short hairpin RNA (shRNA) on ALL cell line (HSB-2) viability. This will help to understand the exact role of CD55 in the pathogenesis of acute leukemia and probably address the utility of anti-CD55 targeted therapy in acute leukemia patients.

## Patients and methods

This is a case–control study which included 60 patients who were diagnosed with de novo acute leukemia at the National Cancer Institute (NCI), Cairo University. All patients were newly diagnosed before any therapeutic interference and showed good performance status with no other comorbidity.

### Assessment of the expression level of CD55 by quantitative real-time polymerase chain reaction (qRT-PCR)

Peripheral blood (PB) samples were drawn from 34 AML patients and 26 ALL patients with ages ranging from 25 to 60 years. Control blood samples were also collected from 30 healthy volunteers within the same age group.

Total RNA was extracted from the whole blood of all subjects using RNA isolation Kit (Applied Biosystems™) according to the manufacturer’s instruction. The purity and the amount of the isolated RNA were assessed using the NanoDrop® (ND)−1000 spectrophotometer (NanoDrop Technologies, Inc. Wilmington, USA). Complementary DNA (cDNA) was synthesized using the Applied Biosystems™ High-Capacity cDNA Reverse Transcription Kit (Thermo Fisher Scientific, cat no. 4368814).

The qRT-PCR was performed using fluorescent TaqMan Gene Expression Assays (ID: Hs00892614_g1, cat. no. 4351372), and *β*-actin was used as the endogenous housekeeping control assay (Thermo Fisher Scientific). The RT-PCR amplification was done using the Applied Biosystems StepOnePlus™ Real-Time PCR System. The instrument was programmed to the standard run according to the TaqMan® gene expression assays protocol. The expression of the target genes was determined by the relative fold change using the 2-ΔΔ CT method [27].

### Transfection of HSB-2 cells with short hairpin RNA (se-35012-SH)

The cancer ALL cell line (HSB-2) was obtained from the American Type Culture Collection (ATCC, Manassas, VA, USA). Serial subculturing was performed and maintained in RPMI-1640 medium (Sigma–Aldrich, USA) supplemented with 1% penicillin/streptomycin and 10% fetal bovine serum (FBS) at 37 °C in a humidified 5% CO2 incubator.

The HSB-2 cells were seeded in a 6-well plate (70–80% confluence) for 48 h before transfection. Customized CD55 shRNA plasmid (h) (sc-35012-SH, Santa Cruz Biotechnology, NC, USA) was used for the inhibition of CD55 expression in human cells. It consisted of a pool of three target-specific lentiviral vector plasmids, each encoding 19–25 nt plus the hairpin. The shRNAs were designed to knock down gene expression. Each plasmid contains a puromycin resistance gene for the selection of cells that were stably expressing shRNA.

Three ShRNA plasmids included the following: (1) the CD55 shRNA plasmid to knockdown CD55 gene expression, (2) combined CD46 and CD55 shRNA plasmids to knockdown both genes and observe the effect of co-silencing of these mCRPs on ALL cells, and (3) the mock shRNA that encoded a scrambled target to eliminate any nonspecific effects that may be caused by the transfection reagent or process. The mCRP expression in HSB-2 cells is not clear; therefore, CD46 was chosen to avoid that other MCRP can compensate for the inhibition of CD55, as both can inhibit C3. The transfection procedure was performed according to the manufacturer’s instructions (Santa Cruz Biotechnology, USA). The shRNA plasmids and their corresponding RNA sequences were illustrated in Table 1.

**Table 1:** The shRNA plasmid sequences and their corresponding RNA sequences

**1-se-35012-SHA**	Hairpin sequence: GATCCCCATCTCCTTCTCATGTAATTCAAGAGATTACATGAGAAGGAGATGGTTTTT
**Corresponding siRNA sequences (sc-35012 A)**	Sense: CCAUCUcCUUCUCAUGUAAtt
	Antisense: UUACAUGAGAAGGAGAUGGt
**2-sc-35012-SHB**	Hairpin sequence: GATCCGGATATAGACAGTCTGTAATTCAAGAGATTACAGACTGTCTATATCCTTTTT
**Corresponding siRNA sequences (sc-35012B)**	Sense: GGAUAUAGACAGUCUGUAAtt
	Antisense: UUACAGACUGUCUAUAUCCt
**3-sc-35012-SHC**	Hairpin sequence: GATCCCTCACCAACTTCTCAGAAATTCAAGAGATTTCTGAGAAGTTGGTGAGTTTTT
**Corresponding siRNA sequences (sc-35012 C)**	Sense: CUCACCAACUUCUCAGAAAtt
	Antisense: UUUCUGAGAAGUUGGUGAGt

All sequences are provided in 5′–3′ orientation

### Assessment of CD55 protein expression post-transfection using flow cytometric analysis

The CD55 protein expression level post-transfection was measured using a flow cytometry assay at a cell count of approximately 10,000 cells. CD55 monoclonal antibody (PE, Thermo Fisher Scientific, MHCD5504) and CD46 monoclonal antibody (FITC, Thermo Fisher Scientific, MA5-49,013) were used to measure the level of both proteins. Isotype control was used to avoid nonspecific binding. Stained tumor cells were analyzed on FACSCalibur flow cytometry (Beckman Coulter, USA) using CellQuest Pro software.

### Cell viability assay

The (3-(4,5-dimethylthiazol-2-yl)−2,5-diphenyltetrazoliumbromide thiazolyl (MTT) assay was performed using human serum freshly prepared from healthy blood donors as a source of complement to assess the cell viability after post-CD55 gene silencing. Briefly, the HSB-2 cells were seeded in a 96-well plate in duplicates in RPMI-1640. The experimental arms of this experiment were formed of HSB-2 cells treated with human serum without CD55 knockout, HSB-2 cells treated with human serum with shRNA for CD55 knockout, HSB-2 cells treated with human serum with shRNA for CD55 and CD46 knockout, HSB-2 cells treated with human serum, and RPMI media only as a blank. After culturing overnight at 37 C, 5% CO_2_ and 90% humidity, the media was removed and replaced with 50 µl of fresh media plus 50 µl of a prepared sterile MTT solution (5 mg/ml PBS) in each well. Then after 4 h, the formed formazan was dissolved in 100 µl of DMSO and measured by a Vector 1420 microplate reader at 595 nm.

### Statistical analysis

Using GraphPad Prism software (Version 5 for Microsoft Windows), the data were presented as mean and standard deviation (SD). The analysis of different groups was done using one-way ANOVA and unpaired *t*-test. All tests were performed 2-tailed, and *p* < 0.05 was considered a significant difference.

## Results

### Patients’ characteristics

The present study included 60 patients diagnosed with AML [18/34 (52.9%) males and 16/34 (47.1%) females] and ALL [18/26 (69.2%) males and 8/26 (30.8%) females]. The mean ages for the included patients were 44.8 ± 12.3 years for AML patients and 46 ± 13.6 years for ALL patients (Table 2).

**Table 2:** Clinical characteristics of the assessed acute leukemia patients

Variables		AML (*n* = 34)	ALL (*n* = 26)	Controls (*n* = 30)	*p*-value
Gender	Male	18 (52.9%)	18 (69.2%)	16 (53.3%)	0.153
	Females	16 (47.1%)	8 (30.8%)	14 (46.7%)	
Age (years)	Mean ± SD	44.8 ± 12.3	46 ± 13.6	45 ± 7.6	0.095
CD55 expression	RQ	0.2499 ± 0.074	0.2581 ± 0.095	1	*P* < 0.001

*RQ* relative quantification

### The expression levels of CD55 in acute leukemia patients

**Fig. 1:**
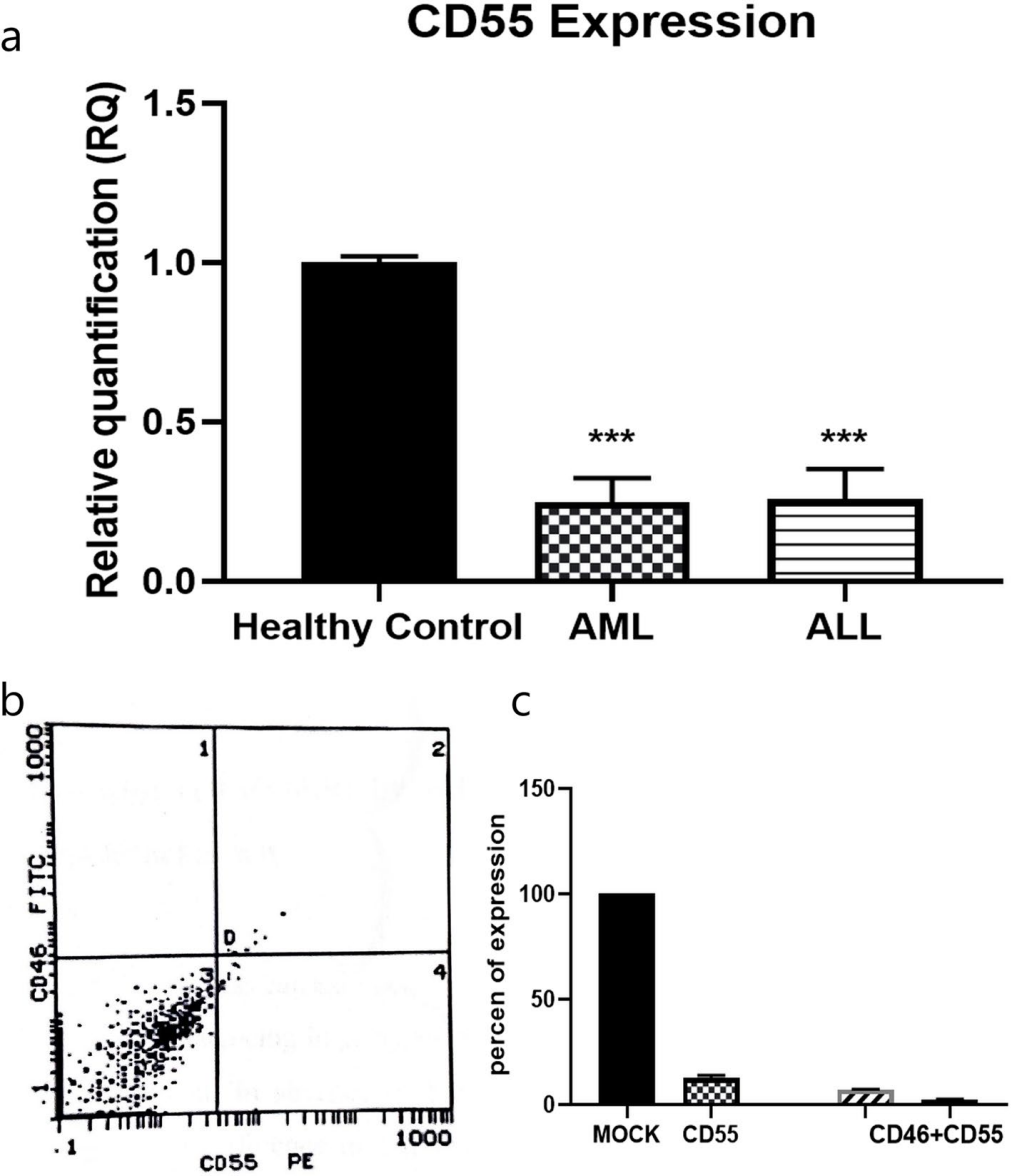
**a** CD55 gene expression in AML and ALL patients in comparison to healthy controls. Significance of CD55 mRNA expression level is represented by *** for *p* < 0.001. **b** Flow cytometry assay of CD55 and CD46 proteins expression levels post-transfection in anti-CD46 + CD55 hRNA co-transfected HSB-2 cells. There was double negative expression for both CD46 and CD55 in the transfected cells. **c** Determination of shRNA-mediated knockdown of CD55 (second bar) and combined CD46 + CD55 expression (third bar) on acute leukemia cell line HSB-2 by the flow cytometry compared to the Mock cells (first bar). The percentage of inhibition was calculated relative to nonsilencing shRNA controls (Mock) (100%). Data are given as mean value and standard deviations of (*n* 23) experiments

The CD55 gene expression level was significantly downregulated in AML patients compared to healthy controls (*p* < *0.001*), with an RQ value of 0.2499 ± 0.07427. Similarly, the expression level of CD55 was significantly downregulated in ALL patients compared to healthy controls (*p* < *0.001*), with an RQ value of 0.2581 ± 0.09467 (Fig. 1a). There was no significant difference (*p* = *0.153*) in the expression level of CD55 regarding the gender in each group (Table 2).

### CD55 expression analysis post-transfection of HSB-2 cells

To confirm the knockdown of the CD55 after shRNA transfection, the CD55 mRNA level was determined to be decreased to 94 ± 3.2% relative to the mock-transfected cells. Additionally, the post-transfection level of CD55 expression by flow cytometry assay in HSB-2 cells transfected with CD55 shRNA plasmid showed a significant reduction of CD55 expression level (1.6 ± 0.213) by 87.3% compared to the mock-transfected cells. Combined knockdown of both CD46 and CD55 resulted in the silencing of CD46 (0.665 ± 0.05) by 99.3%, and CD55 was silenced by 80.2% (2.5 ± 0.88) in comparison to the mock-transfected cells (Fig. 1b, c).

### Assessing cell viability by MTT assay following posttranscriptional CD55 gene knockdown

**Fig. 2:**
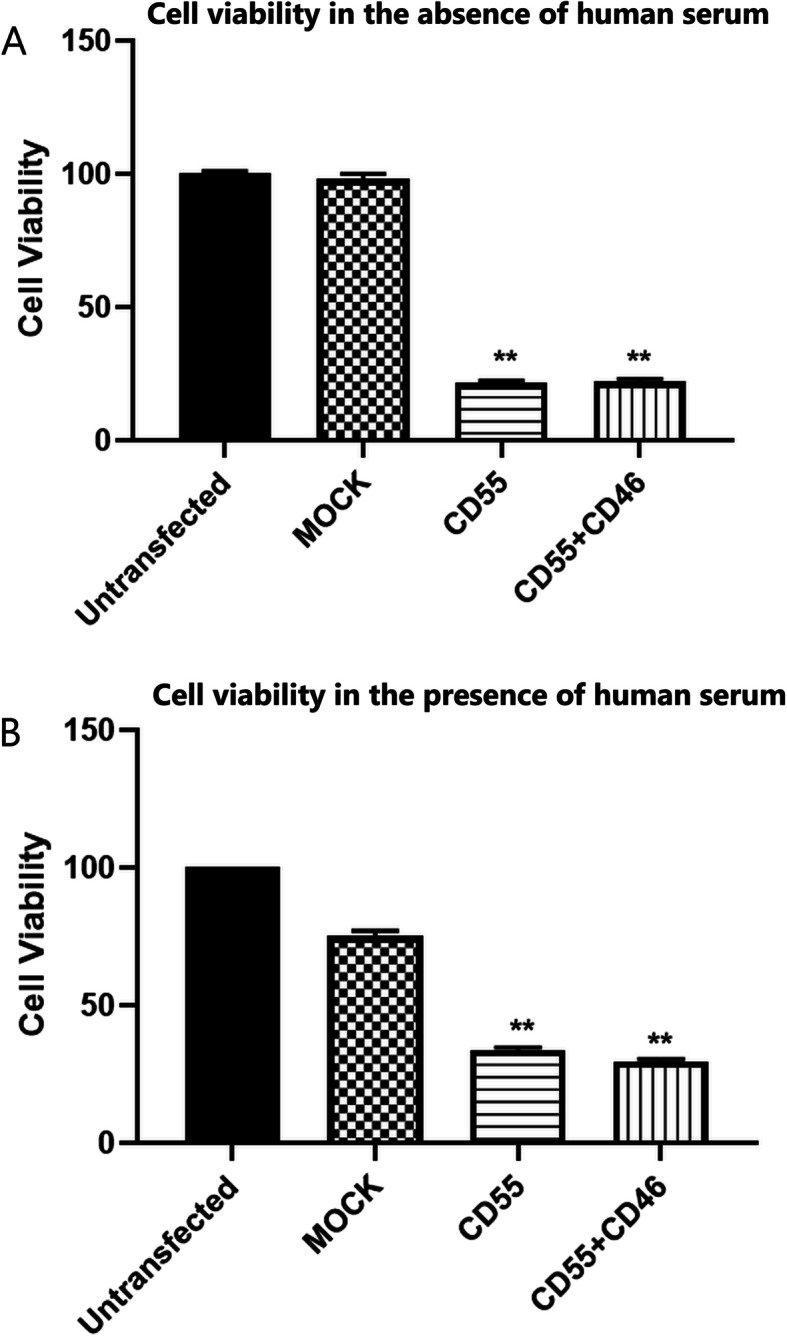
Viability of CD55 shRNA-transfected cells and cells transfected with both CD55 and CD46 silencing shRNA plasmids: **A** In the absence of human serum (*p* < 0.001) and **B** in the presence of human serum (*p* = 0.01). ***For significant *p*-value compared to non-transfected or mock-transfected cells

MTT assay was carried out to assess HSB-2 cells viability post-transfection with shRNA for CD55 silencing in the presence and absence of normal human serum (NHS) as a source of complement. In the absence of serum, the MTT assay results showed that there was no significant difference in cell viability between untreated and mock cells. The cell viability was significantly reduced upon CD55 knockdown by 78.6% as compared to either non-transfected or mock-transfected cells (*p* < 0.001). Also, there was a significant decrease (*p* < 0.001) in cell viability upon co-transfection with both CD55 and CD46 silencing shRNA plasmids in comparison to mock (78.3%) and non-transfected controls (78%, Fig. 2A).

While in the presence of human serum as a source of complement, a significant reduction in cell viability was observed in cells that have been transfected with CD55 silencing shRNA, where cell viability decreased to 66.3% as compared to non-transfected controls. In cells co-transfected with CD55 and CD46 silencing plasmids, cell viability decreased to 70.6% as compared to non-transfected cells (*p* = 0.01). There was no significant difference in cell viability between cells with CD55 silencing and cells with both CD55 and CD46 silencing shRNA (Fig. 2B, Table 3).

**Table 3:** Assessing cell viability by MTT assay following posttranscriptional CD55 gene knockdown

	**Non-transfected**	**MOCK**	**CD55**	**CD55 + CD46**	***p*-value**
In the absence of human serum	99 ± 0.1%a*	98 ± 1.1%a	21.4 ± 3.5%b	21.7 ± 4.6%b	***p* < 0.001**
In the presence of human serum	99 ± 0.09%a	85 ± 8.3%a	33.7 ± 6.2%b	29.4 ± 5.8b	**0.010**

^*^Variables with different letters are significantly different

## Discussion

The complement system’s recognition of tumor cells has been commonly related to an effector activity that leads to the elimination of cancer cells. Consequently, researchers have designed various strategies to enhance complement activation as a mean of developing immunotherapy against tumors [28]. On the other hand, previous studies suggested that the complement might have a tumor-promoting role in mouse models [9, 29]. The idea that the complement elements can act as a cancer promoter is consistent with the tumor immuno-editing theory [30]. The tumor immuno-editing is a process in which the immune system can promote the tumor development through three dynamic phases (3Es). These phases include elimination, equilibrium, and escape. At first, the innate and adaptive immune response can identify and destroy tumor cells efficiently (elimination). However, some tumor cells can evade the immune surveillance and enter in a dormancy phase (equilibrium phase). These escaped cells undergo genetic and epigenetic alterations due to the selection pressure induced by the immune response. After a relatively long period, these immunologically edited cells grow and proliferate to form cancer which can survive and escape the immune surveillance [31]. According to this context, tumor cells could use the convenient balance generated between complement activation and inhibition to their advantage. Also, inflammation is found to have a prominent role in several phases of carcinogenesis and cancer progression [32]. Additionally, the adaptive immune response is controlled by complement activation and could have a role in controlling T-cell response to cancers [33]. Moreover, immune cells, such as macrophages, have both pro- and anticancer phenotypes [34].

The results of the current study indicated that the complement system may have a dual action in cancer. The expression of CD55 was significantly downregulated by approximately 2- to 2000-fold in AML patients and by about 2- to 30-folds in ALL patients compared to normal control. Similar findings were reported in an earlier study done by Guc et al., and that surface expressions of CD55 were significantly lower in ALL and marginally lower in AML patients in comparison to their normal control [35]. Consistently, Bharti et al. concluded in their review that CD55 was found to be highly expressed in chronic myeloid leukemia (CML) and chronic lymphocytic leukemia (CLL); however, its expression in other leukemia types including AML and ALL is still not definitive [18]. Seya et al. also reported that lymphoid cells, especially non-Hodgkin’s lymphoma cells, lacked CD55 expression than other types of hematological malignancies, while the other phosphatidyl inositol-anchored protein CD59 was expressed in most cases [36].

In another study done by Fukuda et al., they concluded that flowcytometric quantitative analysis of the CD55 surface expression showed that two out of eight lymphoid cell lines lacked DAF expression, while no myeloid cell lines lacked it [37]. These data revealed that DAF disappears especially in some lymphoid cells during malignant processing, which could be related to some genomic disorders that take place during malignant transformation [37]. The study also mentioned that these abnormalities of cell-associated mCRPs may render some leukemia cell lines relatively complement-sensitive [37]. Despite the multifactorial role of complement in several disease models, little is known regarding its direct implication in the regulation at the tumor-specific setting [38]. In line with these data, Loeff et al. [39] reported that the expression levels of mCRPs CD55 or CD59 could not be used as a single marker to predict the sensitivity of B-ALL cells to antibody-induced complement-dependent cytotoxicity.

On the other hand, the present results of the MTT assay showed that there was a significant reduction in HSB-2 cell viability upon silencing of the CD55 gene using shRNAs compared to the non-transfected control in the presence of human serum (as a source of complement proteins). These results suggested that CD55 may be involved in the protection of these tumor cells against complement-mediated attack at some point of time. These data are consistent with that reported by Geis et al. that posttranscriptional gene knockdown of CD46, CD55, and CD59 resulted in increased sensitivity of malignant cells to complement lysis [40]. Another explanation could be added that CD55 may work through other pathways independent of complement regulation [18].

Indeed, the current study emphasizes the dual action of the complement system during the tumor development. These data are supported by the in vitro results, which showed the important protective role of the complement elements against tumor progression. However, the results obtained from the AML and ALL patients revealed that CD55 has an inhibitory effect on tumor cell development. This indicated that cancer could evade complement-inflicted damage and manipulate it to provide an amiable microenvironment for cancer to progress and thrive through the cancer immune-editing theory [39]. Moreover, the low expression of CD55 in leukemia patients might be a result of an attempt by the body to make the cancer cells more susceptible to complement-induced damage. However, other complement regulators may compensate for this downregulation, and thus, the cells manage to thrive. Therefore, other mCRPs should be assessed to address the full picture.

Overall, it was suggested that the expression of CD55 decreases complement-mediated cell lysis in tumors, and a lack of CD55 increases the overall inflammatory response [41]. Local complement activation inside tumors raises the question of whether complement activation is pro-cancer or anti-cancer. This is quite similar to the complex part that inflammation plays in cancer. While inflammatory cells and cytokines are significant in immune surveillance, chronic inflammation stimulates carcinogenesis and cancer development. Even with this complex picture, the majority of evidence indicates that chronic inflammation has a pro-cancer effect [33].

Therefore, the multifunctional properties of the complement system implicate it in opposing roles in cancer, where its biological functions are much more diverse than a simple elimination of target cells. Indeed, many factors can be involved to formulate the final picture either to destroy or promote cancer cells. These factors include that other mCRPs could compensate for the downregulation of CD55 and inhibit the complement system. Another possibility is that CD55 can activate or suppress intracellular signalling independent of complement regulation. This leads us to the agreement that further assays must be carried out for further elucidation of the correlation between CD55 and acute leukemia. Also, it is recommended to study the effect of combining CD55 with other mCRPs together such as CD59 on cancer progression. Research should be conducted on the role of CD55 in other types of leukemia such as chronic myeloid, chronic lymphocytic leukemia, and non-Hodgkin lymphoma or other forms of solid tumors.

In conclusion, CD55 was significantly downregulated in acute leukemia. However, its in vitro silencing showed significant reduction in cell viability, giving it a dual opposing role in cancer pathogenesis.

## Data Availability

Data is provided within the manuscript.
